# Spatiotemporal dynamics and forecasting of public attention to entrepreneurship education: An entropy-based modeling approach

**DOI:** 10.1371/journal.pone.0326635

**Published:** 2026-04-10

**Authors:** Xianhang Xu, Hong Liu, Mengjiao Zhao, Mohd Anuar Arshad, Yugang Jian, Shuxia Cao, Guoyu Luo, Qianqian Chen

**Affiliations:** 1 School of Digital Economy and Management, Chongqing Institute of Engineering, Chongqing, China; 2 School of Management, Universiti Sains Malaysia, Penang, Malaysia; 3 School of Economics and Management, Tianjin Tianshi College, Tianjin, China; The University of Warwick, UNITED KINGDOM OF GREAT BRITAIN AND NORTHERN IRELAND

## Abstract

Entrepreneurship education is increasingly important with the development of digital economy and post-pandemic. However, the distribution of public attention to entrepreneurship education (PAEE) remains unclear. Based on Baidu Index data from 2016 to 2024, this study first explores its spatiotemporal patterns. Then, an analytical framework based on Shannon and Tsallis entropy is established to analyze the spatial patterns, and a multi-model forecasting system combining SARIMA, LSTM, XGBoost, and other models is developed. The results show that PAEE shows a trend of continuous decrease, and the concentration in eastern provinces is stronger. Overall, hybrid models generally outperform single models, while the entropy-weighted ensemble demonstrates competitive performance by enhancing robustness and stability. The results of this study can provide quantitative reference for improving policies and promoting fair allocation of regional resources in entrepreneurship education. Meanwhile, this study offers a replicable framework to analyze patterned social behavior and forecasting trends in other fields.

## 1. Introduction

Entrepreneurship education is very important to promote entrepreneurial intentions, improve entrepreneurial ability, and cultivate young talents [1]. In recent years, many countries have been actively promoting the construction of entrepreneurship education ecosystems [2]. It aims to serve and promote young people, stimulate social innovation, enhance employment security, and promote sustainable economic growth [3,4].

After the pandemic, with the global economic transformation and rising employment pressure on young people, entrepreneurship education has continued to expand through digital and collaborative approaches [5]. Meanwhile, it has gained more attention from the public all over the world [6]. Public attention reflects people’s understanding and response to entrepreneurship education. It can serve as an external indicator to evaluate the effectiveness of education development. In turn, it will affect the formulation of policy and allocation of educational resources [7,8].

Recently, many scholars believe that online search data can be used to reflect public attention and track social dynamics [9,10]. However, most of the research on entrepreneurship education only focuses on the internal structure of education, such as curriculum design, teaching innovation, learning assessment, and policy [11–13]. Most of these studies adopt a supply-side perspective or focus on the behavior of organizations in universities. They rely on a relatively static analytical perspective [2]. There is less attention on the temporal and spatial patterns and forecasting models of public attention to entrepreneurship education (PAEE). Specifically, it is necessary to quantitatively examine whether PAEE is evenly distributed or concentrated in certain regions. In addition, it is also necessary to analyze its changing patterns and trends through data-driven forecast frameworks.

However, existing forecasting studies often emphasize statistical accuracy while paying limited attention to whether forecasting results are actionable for policy formulation and educational governance. Recent research highlights that the value of forecasting lies not only in predicting future trends, but in generating stable and interpretable signals that can inform timely and effective decision-making under uncertainty [14]. From this perspective, forecasting PAEE should be understood as a decision-support tool rather than a purely predictive exercise.

Entropy provides a useful way to analyze how public attention changes and how unevenly it is distributed in time and space [15]. Shannon entropy can be used to describe the degree of balance of such attention [16]. Tsallis entropy reflects nonlinear and concentration patterns [17]. These two measures form the foundation for both the analytical and forecasting components of this study.

To address gaps in identifying regional disparities and forecasting attention trends, this study examines PAEE in China using Baidu Index data. Grounded in entropy theory, it develops an integrated research framework combining spatial analysis and forecasting models to explore the spatiotemporal evolution and future changes of PAEE. The research centers on three research questions (RQs):

*RQ1:* What are the temporal trends of PAEE at both yearly and monthly scales?

*RQ2:* How are the spatial patterns of PAEE measured and explained through spatial entropy?

*RQ3:* How can a forecasting framework be developed to improve the accuracy and robustness of PAEE trend analysis?

This study develops a framework that can be used to study public attention in different social settings. We make the following three contributions. First, we introduce public attention as a demand-side indicator of how people respond to entrepreneurship education policies, which is data-driven and interdisciplinary to educational research. Second, we apply entropy theory to measure the spatial patterns of public engagement and thus provide new evidence and policy support about regional disparities in education. Finally, we develop a practical framework that integrates spatial analysis and forecasting models to support decision-oriented analysis of PAEE by emphasizing stability and robustness rather than short-term predictive accuracy.

## 2. Literature review

### 2.1 Evolution and value of entrepreneurship education

Entrepreneurship education originated from the end of the 20th century and has become an important way to cultivate individual creativity and entrepreneurial ability to serve sustainable economic and social development since then [18]. Therefore, it has attracted more and more attention from scholars and policymakers all over the world. Most scholars believe that entrepreneurship education could enhance students’ motivation and entrepreneurial capacity [19]. It also encourages innovation, enhances social mobility, and promotes personal development [20].

Early studies mainly focused on curriculum design, teaching methods, and learning outcomes evaluation, reflecting the supply-side reform [21]. Recently, the range of entrepreneurship education has been broadened from the traditional curriculum design and teaching methods to multiple fields such as interdisciplinary cooperation, digital learning platform, and the analysis of learning behavior [6,13]. Some scholars began to stress that teaching methods should conform to learning objectives. Some scholars believe that entrepreneurship education could be divided into cognitive, skill-based, and practical types according to the learning stages that serve social needs [22]. Meanwhile, more and more attention has been paid to teacher competence and sustainable education environment, which reflects a gradual shift towards integrated and ecological research [23].

Entrepreneurship education has multiple forms of value. From an economic perspective, it motivates young people to start businesses and promotes the development of regional economy [24]. From a social perspective, entrepreneurship education promotes entrepreneurial ethics and sustainability and enables students to develop responsibility and systematic thinking in their entrepreneurial practice [25]. From a psychological perspective, it could improve confidence, creativity, and tolerance to risk [26]. In recent years, the latest research suggests that entrepreneurship education is not limited to skill training. However, it is a long-term educational practice that shapes entrepreneurial personality and social values [27], which serves the social development and institutional support [28].

Although some progress has been made in the supply structure and teaching innovation of entrepreneurship education, current research still ignores the public understanding and response to entrepreneurship education. As an indicator of external effectiveness of educational policies, public attention has rarely been studied in a systematic way and combined with other disciplines. This study attempts to place entrepreneurship education in a broader context of public cognition, and then explores how public attention has changed over time and how it is distributed across regions.

### 2.2. Public attention to educational issues

In a digital society, public attention is an important indicator of social awareness of educational issues [29]. Based on easily measurable online behavior data, such as internet searches and social media use, some scholars reconstructed public cognition based on digital traces [30]. This provides new insights into understanding educational evaluation and policy effects [31].

Different types of digital data may be available, and search engine information, such as Google Trends and Baidu Index, has been found to be useful because it possesses characteristics of wide coverage, time continuity, and geographic detail [9]. Based on these digital data, the change of public concern can be traced, and the differences of regional interest in education can be compared [32–35]. In previous studies, the occurrence of peaks in search engines was found to be associated with major policy releases, exams, or other key educational events [36–38].

Search data analysis also found that different regions have different levels of concern for education, indicating that people’s awareness of educational opportunities and reforms are not uniform [39]. Such findings highlight the potential of digital platforms for understanding how educational information circulates and how public opinions are shaped [40].

Despite a growing body of research using digital data, few studies have focused specifically on PAEE. Most of the existing studies either focus on descriptive accounts or case-based research. There seems to be limited effort into developing temporal patterns or spatial structures. Integrating big data techniques into education research could help reveal how public perception develops over time and how well the communication of relevant policies has been delivered.

### 2.3. Entropy theory application and forecasting model

Entropy is a widely used measure of the heterogeneity of observations in many applied sciences [14]. In spatial analysis and geographic modeling, entropy measures the balance across space, characterizes the complexity of a system, and describes patterns of structural evolution as it develops spatial and temporal heterogeneity. Because of these potential applications, entropy is a useful method to examine regional differences and agglomeration effects [41]. A higher entropy value corresponds to a more uniform distribution of spatial units, while a lower value implies that the arrangement of spatial objects is more concentrated and thus more polarized [42].

Shannon entropy is the most fundamental and widely used entropy measure [43], and has been widely used in fields of physics, ecology, urban studies, and social sciences [44–46]. This breadth of research shows the potential advantages of using entropy to both detect structural differentiation and measure spatial balance. Tsallis entropy is a generalized extension of Shannon entropy. It uniquely introduces a non-additive parameter *q*, which can better capture phenomena related to long-tail distributions, non-stationarity, and nonlinear agglomeration [47]. It has also shown good application prospects in urban expansion, ecosystem evolution, and commercial economy [17,43,48].

Recently, entropy theory has been gradually applied to research on educational evaluation and digital communication because entropy is able to describe the complexity of structural systems while having low model-dependence [49,50]. Spatial entropy is useful for identifying structural components of evaluated regions rather than simply outlining observable regional phenomena. Thus, as it relates to education studies, spatial entropy can help to quantify uneven educational resources and the public’s cognitive aggregation [51,52], which provides a basis for describing public attention and regional polarization.

Public attention is a time series subject to evolving behaviors represented by non-stationarity, periodicity, seasonality, and abrupt changes [53]. However, traditional linear prediction models are often difficult to fully fit owing to the complexity of the changing trajectory. Researchers have increasingly employed a range of time series models and machine learning methods for trend modeling and short-term predictions [54], such as seasonal autoregressive integrated moving average (SARIMA) and theta model, and ensembles (i.e. XGBoost and LightGBM), which are more suited for modeling nonlinear relationships [55,56]. Deep learning models, such as long short-term memory (LSTM) networks, have recently been employed because of their ability to learn and model long-term dependencies and changes [57].

The above methods are beneficial in terms of structure fitting capability and error control. For instance, the SARIMA model handles stationary series with periodicity with persisting lags. The Theta model has better performance when employed for short-to-medium term trend dominated are considered [58]. As integrated learning models, XGBoost and LightGBM models have good nonlinear modeling capability and efficiency [55,56]. Meanwhile, LSTM neural networks are able to effectively learn long-term dependency characteristics in time series [59].

However, a single model has limitations when considering generalizability and model stability. Some researchers have integrated SARIMA, Theta, and other traditional models with LightGBM and LSTM models for the residuals in order to improve overall predictive performance [60,61]. Entropy-weighted method has further been introduced to aggregate forecasting results in a principled manner, thereby enhancing model stability and robustness [62–64]. Taken together, prior studies suggest that regression toward stable averages reduces noise and improves forecast performance [65], while related research emphasizes that stability-oriented forecast combination enhances reliability over short-term accuracy gains [66].

In summary, spatial entropy provides a theoretical framework for measuring the spatial structure of PAEE, while the entropy-weighted method gives a technical approach for integrating multiple forecasting models. Using entropy theory helps explain the evolution of PAEE and improves the clarity and flexibility of the forecasting model.

## 3. Methodology

Grounded in spatial entropy analysis and entropy-weighted method, this study examines the spatiotemporal distribution of PAEE and forecasting framework. It is conducted in three stages: data collection and preprocessing, spatial entropy analysis to evaluate structural complexity and regional differences, and multi-model forecasting with entropy-weighted method to enhance prediction accuracy and robustness.

### 3.1. Data source

The data were collected from the Baidu Index platform (https://index.baidu.com), based on the keyword “entrepreneurship education.” They represent the level of public search interest for 31 provinces in China from January 2016 to December 2024. Hong Kong, Macao, and Taiwan were excluded due to data limitations. All the data used in this study came from public databases and did not involve ethics.

The data reflect the changes in public attention after the implementation of the “mass entrepreneurship and innovation” (Shuangchuang) policy and the impact of the COVID-19 pandemic. To avoid possible bias, the Baidu Index values of zero were replaced by 0.01, so that the regions with lower attention can be included in the spatial analysis.

As the Baidu Index data reflect only the online behavior of Chinese internet users, the reflected characteristics are mostly the features of China’s people and internet environment. Although the analytical framework in this study is reproducible and can be applied to other regions with similar data sources, the results of applying it to other regions should be interpreted with caution.

### 3.2. Spatial entropy analysis

#### 3.2.1. Shannon entropy.

Shannon entropy is applied to quantify the spatial distribution of PAEE across provinces.


$$H=-\sum_{i=\text{1}}^{n}p_{i}\;{\text{log}}_{\text{2}}p_{i}$$ (1)

$$H_{norm}=\frac{H}{{\text{log}}_{\text{2}}(n)}$$ (2)

Where *pᵢ* is the share of PAEE in the *i-*th province, *n* is the number of provinces, and *Hₙₒᵣₘ* is the normalized entropy. The larger *Hₙₒᵣₘ*, the more evenly distributed PAEE is over provinces; the smaller *Hₙₒᵣₘ*, the more concentrated in a few provinces.

#### 3.2.2. Tsallis entropy.

To capture the spatial concentration of public attention more clearly, this study uses Tsallis entropy as another measure. Tsallis entropy is more sensitive to high-frequency and extreme values than Shannon entropy. It is suitable for identifying structural changes in regional dominance [47].


$$H_{q}=\frac{\text{1}}{q-\text{1}}\left(\text{1}-\sum_{i=\text{1}}^{n}\overset{q}{\underset{i}{p}}\right)$$ (3)

Here, *q* is a parameter that controls the sensitivity of the corresponding entropy measure to extreme distributions. When *q* approaches 1, Tsallis entropy coincides with Shannon entropy. When *q* > 1, it puts more weight to dominant regions in the spatial structure [17]. In this study, we set *q* = 2 to show how response of system to spatial polarization.

#### 3.2.3. Evolution Trend and Significance Test.

To analyze the yearly changes of spatial entropy from 2016 to 2024, we drew the line charts of Shannon and Tsallis entropy, and used simple linear regression to assess whether the trend was significantly increasing or decreasing. Take the slope direction and *p*-value to determine whether there was a clear centralization trend of spatial structure.

#### 3.2.4. Spatial Visualization.

Use Python to draw spatial entropy heat maps and time series trend plots to visualize the change of spatial structures over time. Based on the yearly entropy term of each province, a provincial heat map was made to show the contribution of different regions to the total spatial entropy. These visualizations can display the main areas with high PAEE and the surrounding areas with low engagement.

### 3.3. Construction of the forecasting model

This study develops a multi-model framework that combines traditional time series, machine learning, and deep learning methods to forecast the trend of PAEE. The five single models are SARIMA, Theta, XGBoost, LightGBM, and LSTM. The hybrid models include SARIMA-LSTM and Theta-LightGBM. Finally, we use entropy-weighted method to integrate their outputs and improve the overall performance.

SARIMA captures linear trends, seasonality, and short-term changes. The Theta model shows medium-term trend. XGBoost and LightGBM handle nonlinear patterns and sudden changes. LSTM captures long-term changes in PAEE. The two hybrid models bring together linear and nonlinear features to make the results more stable and reduce bias. The entropy-based method gives each model a fair weight, focusing on stability, accuracy, and stronger forecasting results.

#### 3.3.1. Single forecasting model.

(1) **SARIMA Model**

The SARIMA model is fruitful for modeling linear trends and seasonal variations. In estimating the order for time series data, the order can be assessed through a stationarity test and ACF and PACF plots, and the fitting and forecasting happen with the chosen optimal combination of SARIMA (p, d, q) (P, D, Q)s [58].

(2) **Theta Model**

The Theta model applies to short-to-medium term trend-driven series forecasting. The Theta model has a simple structure and calculations to implement quickly. This model uses trend amplification and exponential smoothing on the original series; hence, it decomposes trend and seasonal effects in the series and ways to fit them in rapid forecasting [57].

(3) **XGBoost Model**

The XGBoost model has strong nonlinear modeling and generalization abilities, making it applicable for sudden changes and complex fluctuations when predicting attention changes. The model builds multi-order lag feature as input and used the gradient boosting regression algorithm to train and predict the public attention series [55].

(4) **LightGBM Model**

LightGBM model is stable for modeling mutation features and nonlinear relationships. It builds multi-order lag feature as input variables and trained the regression model from historical attention and modifies to capture complex time fluctuation trends. It helped in fitting better with the complex trend fluctuations employing its efficient mechanism to split features by learning advantages of parallel computing process, making it suitable for short-term prediction and error correction success [56].

(5) **LSTM Model**

LSTM model has been used to get through the nonlinear characteristics and long-term dependencies problems among public attention data. The model is built by using the sequence sliding window approach, to extract input features which use multi-order lagged features, trained an end-to-end neural net, to characterize the dynamic changes of the sequence for large trend predictive processes [59].

#### 3.3.2. Hybrid forecasting model.

(6) **SARIMA-LSTM Model**

The model realized the power of the SARIMA linear and seasonal approach to traditional time series modeling, and the power of the deep learning approach for pattern recognition and relationships. First, the attention was fitted and predict linear and seasonal structure through the SARIMA. Next, the prediction residual was extracted for use as input into the LSTM neural network. Hence, the model could optimally model the series attention trends not only in a single source approach but through multi-level model with combining the attention from SARIMA and LSTM structure [67].

(7) **Theta-LightGBM Model**

In this model, time series trend modeling and nonlinear error modeling was considered into one model. The Theta model extracts and predicts the linear trend and seasonal components from series. Finally fitting the residuals, through LightGBM, enables both models’ collaborative attention at a time. The use of recursive correction increases overall forecasting accuracy, and improves the model in the adaptability to the complex fluctuations of series attention data [61].

#### 3.3.3. Entropy-weighted model.

To increase the forecast stability and accuracy of the model, this study applies the entropy-weighted method to weight the output results of all forecasting models for fusion [68]. This research uses entropy-weighted method as an objective weight assignment method, utilizing the discreteness of forecasting results for each model as the way to assign weights [69]. In this way, we can leverage the advantages of prediction from each of the models, mitigate the influence of bias from a singular model, and better represent a reliable final output value [70]. To ensure a leakage-free evaluation, the entropy-based weights were determined exclusively using information from the training period (January 2016–December 2023). The evaluation period (January–December 2024) was strictly excluded from the weight estimation process. Steps include:

(1) Forecasting matrix construction. Collect the forecasting values of *i* months for *t* models generated within the training period to create forecasting matrix Y = [𝑦ᵢₜ].
(2) Use an inverse error mechanism to normalize the data values and obtain a normalized matrix, Z = [𝑧ᵢₜ]. The equation is:

$$z_{it}=\frac{\text{1}}{\text{1}+\left|y_{it}-\overset{actual}{\underset{i}{y}}\right|+\varepsilon }$$ (4)

Here, ε = 10⁻¹⁰ to ensure the denominator is not zero.

(3) Derive the weight and information entropy for each model based on training-period forecasting errors only. The equations are:


$$P_{it}=z_{it}/\sum_{i=\text{1}}^{I}z_{it}$$ (5)

$$e_{t}=-\frac{\text{1}}{lnI}\sum_{i=\text{1}}^{I}P_{it}lnP_{it}$$ (6)

(4) Derive the weight for each model. The equation is:


$$w_{t}=\frac{\text{1}-e_{t}}{n-\sum_{t=\text{1}}^{n}e_{t}}$$ (7)

(5) Derive the weighted average of the forecasting values for each model to achieve the value of the fusion model. The resulting weights were fixed prior to evaluating the 2024 data, and the final ensemble forecasts were obtained as:


$$\hat{y_{t}}=\sum_{t=\text{1}}^{n}w_{t}y_{it}$$ (8)

#### 3.3.4. Model evaluation.

Once the model was constructed, root mean square error (RMSE) and mean absolute percentage error (MAPE) to assess the forecasting performance of all the models and determine the threshold for the overall evaluation were systematically compared, as well as directional accuracy (DA) and the determination coefficient (R^2^). RMSE and MAPE measure the magnitude of error, while DA evaluates directional consistency and R^2^ reflects goodness of fit [71]. All performance metrics were computed using the 2024 dataset, which was not involved in model training or entropy-weight estimation, thereby representing a genuine out-of-sample forecasting evaluation.

## 4. Results

### 4.1. General trend of public attention to entrepreneurship education

**Fig 1:**
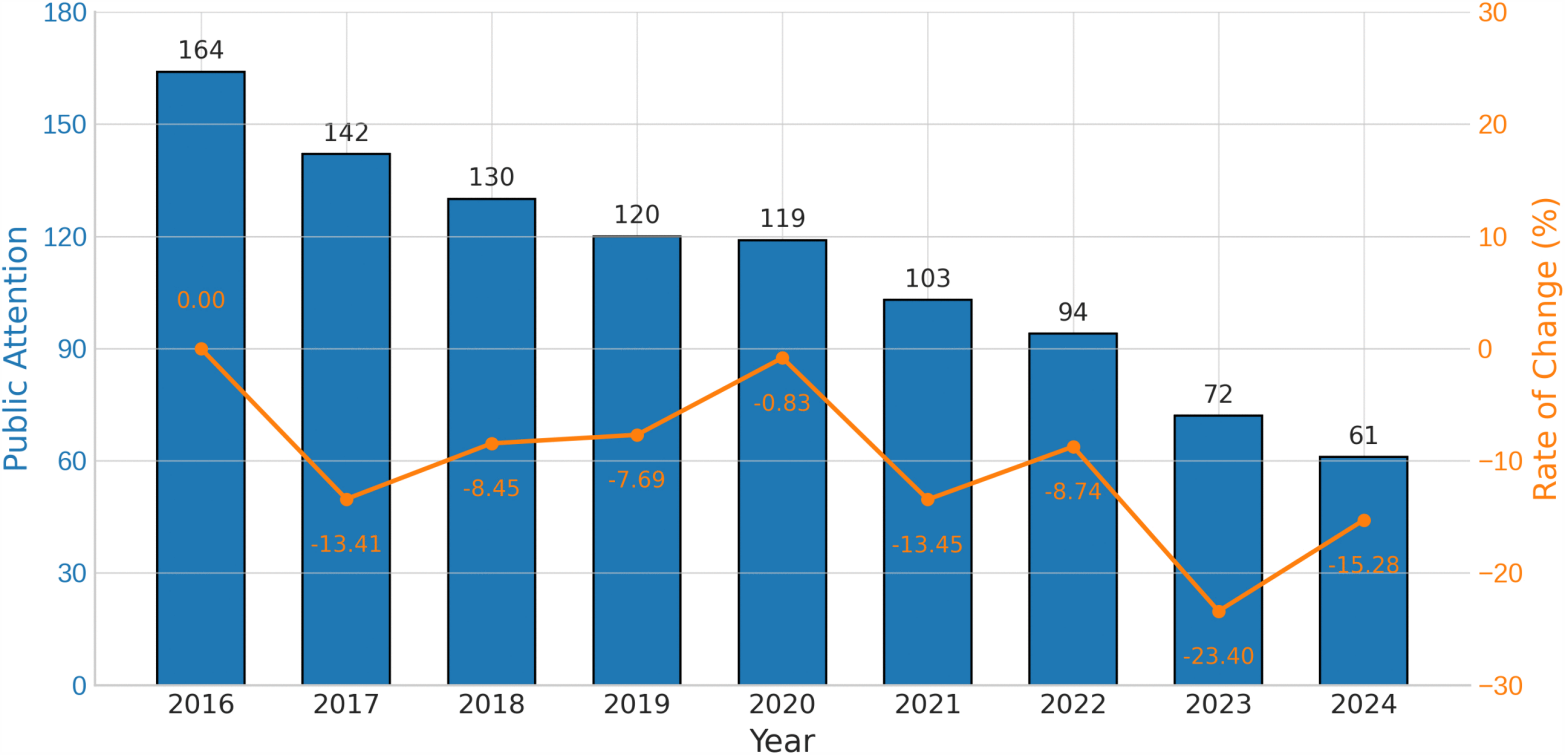
Yearly Public Attention to Entrepreneurship Education in China (2016–2024).

According to Fig 1, the PAEE in China has remained in an overall downward trend from 2016−2024. The PAEE in 2016 was 164, the highest value in this phase. It fluctuated over the years and declined to 61 in 2024, representing a 62.8% decrease. The largest year-on-year drop occurred in 2023 (−23.40%), followed by 2021 (−13.45%) and 2017 (−13.41%). Interestingly, the smallest decrease occurred in 2020 at −0.83%.

The average PAEE value over the nine-year period was 111.67 and the standard deviation was 32.87, reflecting considerable overall variation. The median was 119, nearly the same as the level in 2019–2020. Although there were short periods of stability, the overall trend was a steady decrease in PAEE. This also shows that we need to make more efforts to maintain the public’s interest in entrepreneurship education.

**Fig 2:**
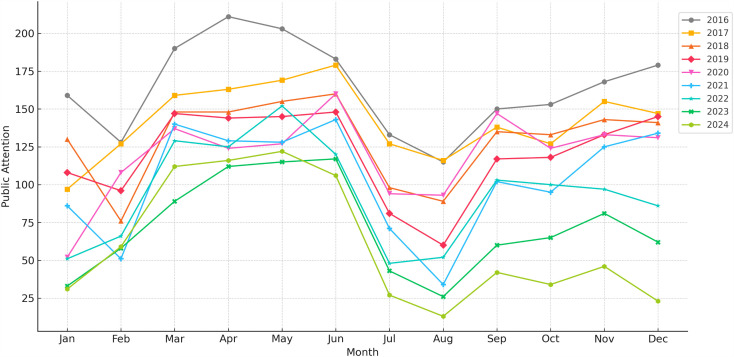
Monthly Public Attention to Entrepreneurship Education in China (2016–2024).

Fig 2 shows that PAEE in China exhibited clear seasonal patterns at the monthly level. The overall pattern follows a “spring rise–summer dip–autumn/winter fluctuation” cycle.

The highest level of PAEE was usually from March to June. This is in line with the school calendar, major entrepreneurship events, and policy releases. In July and August, the values dropped sharply. August had the lowest average value in a month, only 66.44. Notably, in December, the standard deviation was 49.25, which means a large swing at the end of the year. These seasonal variations show obvious seasonal variation, which can help plan entrepreneurship education initiatives.

Ordinary least squares (OLS) regression was employed to analyze the annual and monthly trends in PAEE in China. The results showed a significant downward trend at both time scales. At an annual timescale, the regression slope was −11.85, which means that PAEE decreased by 11.85 Baidu Index units annually (R^2^ = 0.974; p = 7.81 × 10 ⁻ ⁷). At a monthly timescale, the average decrease was 0.98 units per month (R^2^ = 0.492; p = 2.85 × 10 ⁻ ¹⁷).

### 4.2. Spatial entropy analysis of public attention to entrepreneurship education

This study employs Shannon and Tsallis entropy analysis, spatial significance tests, and provincial entropy heat maps to show the spatial distribution of PAEE at national and regional levels.

#### 4.2.1. Shannon entropy analysis.

**Fig 3:**
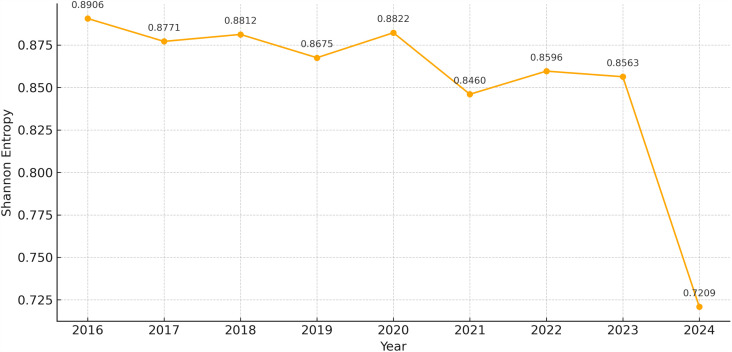
Shannon Entropy of Public Attention to Entrepreneurship Education in China (2016-2024).

Shannon entropy for PAEE was calculated for 31 provinces in China from 2016 to 2024 and then normalized to make the results comparable across years. The values show a steady decline, indicating that PAEE has become less balanced and more concentrated in a few regions (see Fig 3).

The normalized Shannon entropy value was 0.8912 in 2016, which means that PAEE was relatively evenly distributed in all regions. In 2024, the Shannon entropy dropped to 0.7209, which is more than 19% lower than that in 2016, indicating that PAEE became more concentrated in fewer provinces. It means that the spatial diversity decreased and the polarization of regions strengthened. The drop after 2021 may accelerate the information exposure gap between regions, which may be caused by policy changes, uneven communication, or public events after the pandemic.

#### 4.2.2. Tsallis entropy analysis.

In order to show the spatial polarization more clearly, Tsallis entropy (*q* = 2) was selected as the local measure. Tsallis entropy gives more weight to the dominant provinces, and its clustering effect is stronger than Shannon entropy.

**Fig 4:**
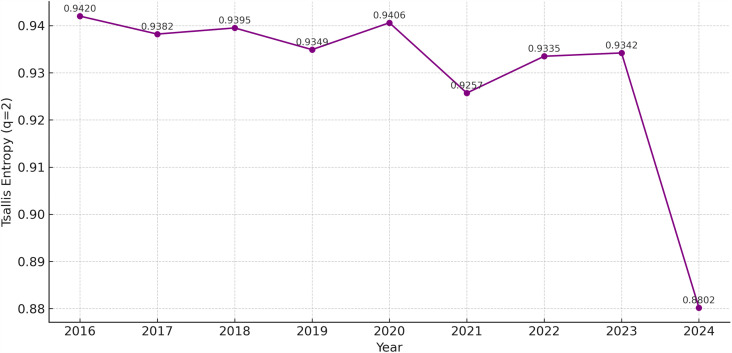
Tsallis Entropy of Public Attention to Entrepreneurship Education in China (2016-2024).

As shown in Fig 4, the results show a similar trend: the values decreased over time. In 2023 and 2024, the value dropped dramatically from 0.9342 to 0.8802. It means that PAEE was more concentrated in a few provinces, and other areas played a smaller role. This suggests that the spatial pattern of PAEE is changing from a relatively balanced layout to a more concentrated pattern around leading provinces.

#### 4.2.3. Significance test of spatial entropy change.

The spatial entropy trend was tested by OLS as shown in Table 1. OLS results show that Shannon entropy showed a downward trend, which means that the PAEE was more concentrated in regions over time, and the regional gaps were widened. Tsallis entropy (*q* = 2) also showed a slight downward trend, but the change was not significant.

**Table 1:** OLS results of spatial entropy trend analysis.

Entropy Type	Slope	*p*-value	R²	Significance
Shannon entropy	−0.0134	0.032	0.506	Significant at 5% level
Tsallis entropy (*q* = 2)	−0.0047	0.051	0.443	Not significant

The two measures show a mixed pattern. Shannon entropy shows that the concentration of PAEE across regions is strengthened, while Tsallis entropy shows that the local PAEE is still very unstable. Generally, the PAEE is clustering in a few provinces, and the regional differences continue to change.

#### 4.2.4. Heat map analysis of entropy items in each province.

**Fig 5:**
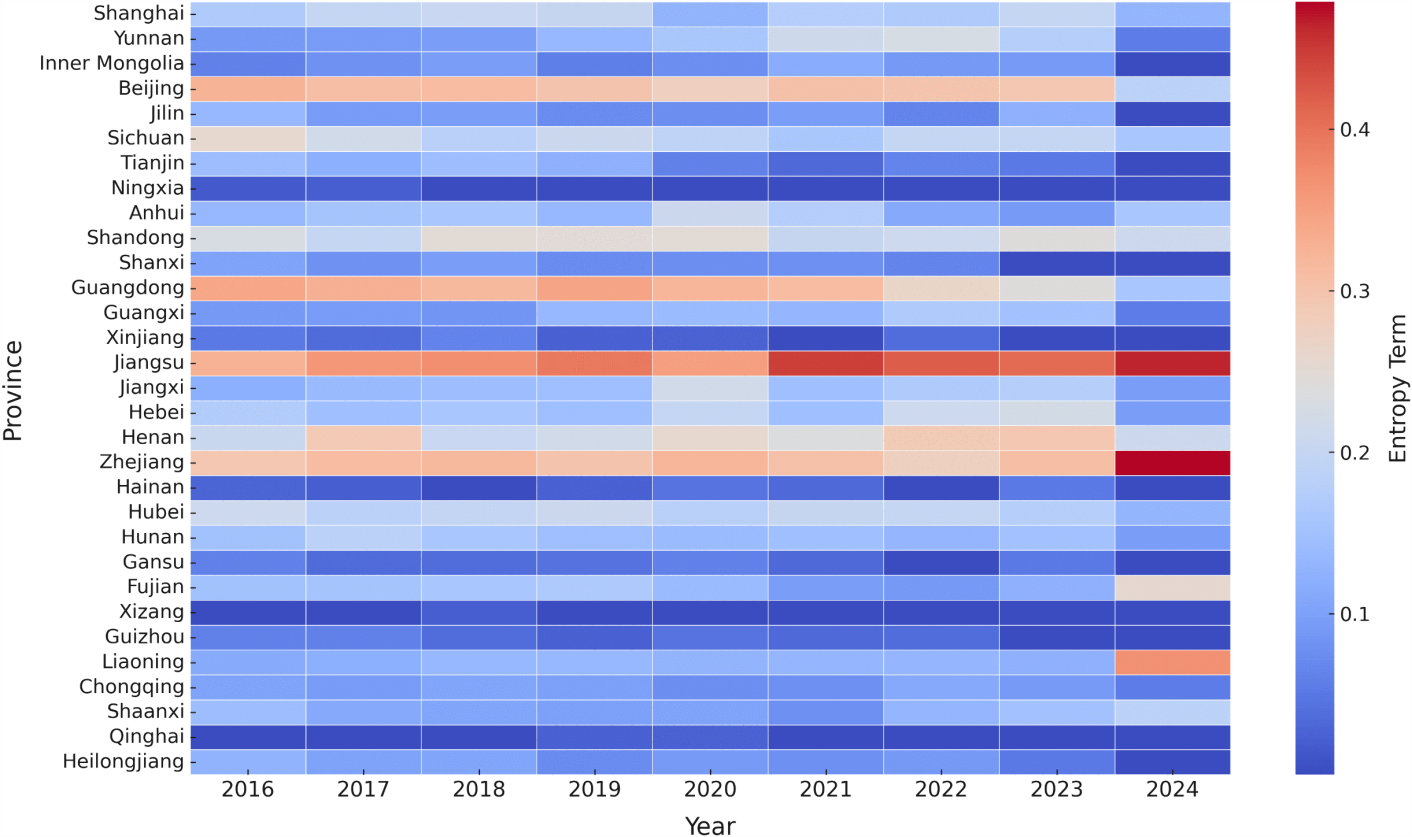
Provincial heat map of shannon entropy in China (2016-2024).

The entropy term of each province was calculated, and then the heat map was drawn to show each province’s contribution to the spatial pattern (see Fig 5).

The heat map shows that the entropy terms of developed eastern provinces such as Jiangsu, Zhejiang, and Guangdong are relatively high, showing these provinces play a steady and important role in the spatial distribution of PAEE. On the contrary, the entropy terms of Beijing and Shanghai declined sharply in 2022, which shows that more PAEE has concentrated in these two cities. While in western and less developed areas such as Xizang, Qinghai, Ningxia, and Gansu, entropy terms remain low and show that these provinces have limited involvement and low levels of PAEE.

Overall, provinces with high entropy terms in the heat map gradually contracted from 2016 to 2020, and the trend of spatial marginalization intensified in 2021–2023. In 2024, however, a local rebound was observed in several provinces, such as Fujian and Liaoning. This suggests that although the spatial structure of PAEE has become increasingly concentrated, there remains potential for rebalancing, driven by policy changes or fluctuations in public discourse in certain years.

### 4.3. Modeling the forecasting of public attention to entrepreneurship education

#### 4.3.1. Forecasting model building.

**Fig 6:**
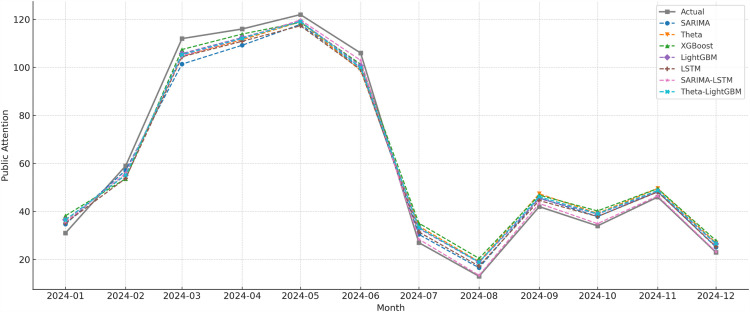
Forecasting results of models.

The data from January 2016 to December 2023 were used as the training set, and the data from January to December 2024 served as the validation set. A total of seven forecasting models including SARIMA, Theta, XGBoost, LightGBM, LSTM, SARIMA-LSTM, and Theta-LightGBM were applied to train and forecast PAEE trends. Fig 6 presents a comparison between the forecasted values for 2024 and the actual observations.

The LSTM model tracks fluctuations well and adapts to dynamic changes. Both the Theta and LightGBM models capture the main trend, though they sometimes fall short in reflecting short-term changes. XGBoost does not respond well to local shifts and often leaves visible gaps from the real values in several months. SARIMA gives a stable curve and captures seasonal changes, but it responds less to complex shifts.

From the trend comparison, hybrid models perform better than the single ones. The SARIMA-LSTM model fits the real data most closely. It handles sharp changes well and shows good performance in following time series patterns. Theta-LightGBM model produces a smooth forecast curve. It reflects the long-term trend well, though it reacts a bit slowly during rapid growth. In short, the hybrid models track trend changes more accurately and identify turning points more clearly, making them more suitable for time series forecasting with both cyclical and nonlinear changes.

#### 4.3.2. Model evaluation.

To assess how well each model forecasts PAEE, four evaluation indicators were used: MAPE, RMSE, DA, and R^2^. Table 2 shows that the models differ clearly in forecasting performance.

**Table 2:** Model accuracy evaluation.

Model	MAPE	RMSE	DA	R²
SARIMA	11.82%	8.95	75.00%	0.84
Theta	13.57%	10.23	66.67%	0.79
XGBoost	14.92%	11.34	58.33%	0.76
LightGBM	12.38%	9.21	66.67%	0.83
LSTM	11.05%	8.67	75.00%	0.85
SARIMA-LSTM	9.73%	7.82	91.67%	0.88
Theta-LightGBM	10.52%	6.23	66.67%	0.90

Among the five single models, LSTM and SARIMA both performed well, particularly in fitting accuracy and trend detection. LSTM showed advantages in capturing sequence changes and gave stable results, while SARIMA worked better in showing seasonal patterns. In contrast, although LightGBM and XGBoost have the ability of nonlinear modeling, they have large fluctuations in actual fitting, low trend judgment accuracy, and perform worse than other models. The Theta model can restore the overall trend well, but it is still insufficient in capturing local changes.

Both hybrid models outperform their respective basic models in forecasting performance. Among them, the overall trend of the SARIMA-LSTM model is closest to reality, with stable trend fitting and accurate direction judgment, reflecting strong dynamic capture ability and nonlinear adaptability. The Theta-LightGBM model performs outstandingly in overall error control and trend consistency, with strong model coordination ability, and is suitable for complex sequences where trend capture and residual correction coexist.

Overall, while traditional single models possess conceptually simple structures, they tend to have lower performance in forecasting a time series, such as in the case of PAEE, as it is periodic, nonlinear, and subject to local volatility. By combining linear trend modeling with nonlinear error correction, the hybrid models handle error better, match the data more closely, and keep the main trend steady. The results demonstrate that hybrid model is an effective method to deal with data with complex patterns.

#### 4.3.3. Entropy-weighted model forecasting.

To make the forecast more reliable, this study uses entropy weighting to combine the results of different models and develops an integrated forecasting model. The method examines the variation of model outputs, and gives different weight according to their contribution. The entropy-based weights were estimated using training-period information only and subsequently applied to generate ensemble forecasts for the 2024 evaluation period. The weights are calculated using equations (4)-(7), and the results are presented in Table 3.

**Table 3:** The weight of models.

Model	Weight
SARIMA	0.1176
Theta	0.1863
XGBoost	0.1061
LightGBM	0.0981
LSTM	0.1292
SARIMA-LSTM	0.2041
Theta-LightGBM	0.1586

Note: *Weights were estimated using training-period data (2016–2023) only.*

The results of each model were weighted, and the final results of entropy-weighted model forecast were obtained. These forecasts were evaluated and compared with the optimal values of other models (see Table 4). Based on the entropy-weighted method, the SARIMA-LSTM model receives the largest weight, followed by Theta and Theta-LightGBM, indicating that hybrid models and trend-sensitive methods contribute more information to the ensemble forecasts.

**Table 4:** Entropy-weighted model forecasting performance analysis.

Indicator	Entropy-weighted model value	Other model optimal values	Improvement
MAPE	10.93%	SARIMA-LSTM（9.73%）	−1.2%
RMSE	4.46	Theta-LightGBM（6.23）	1.77
DA	100%	SARIMA-LSTM（91.67%）	8.33%
R²	0.99	Theta-LightGBM（0.90）	0.09

Note: *All performance metrics were computed on the 2024 out-of-sample evaluation period.*

The results show that the entropy-weighted model demonstrates competitive overall performance when compared with both single and hybrid models across multiple evaluation metrics. In terms of relative error, the entropy-weighted approach achieves a lower MAPE than all single models. However, its MAPE remains slightly higher than hybrid models. This suggests that while entropy-based averaging improves robustness by reducing extreme percentage errors, it does not fully capture the structural advantages embedded in hybrid modeling frameworks. RMSE decreased to 4.46, which indicates that the fitting results are smoother and more accurate. As for trend following, entropy-weighted model maintained the best directional accuracy, which means that entropy-weighted model has high sensitivity and directional stability. R^2^ increased to 0.99, which means that entropy-weighted model has stronger explanation ability.

The above results show that entropy-weighted model enhances forecasting stability and robustness, whereas hybrid models that explicitly integrate structural and nonlinear components remain more effective in minimizing relative errors and modeling complex temporal dynamics. Therefore, the entropy-weighted model should be regarded as a complementary approach rather than a replacement for hybrid forecasting models when dealing with highly fluctuating PAEE data.

**Fig 7:**
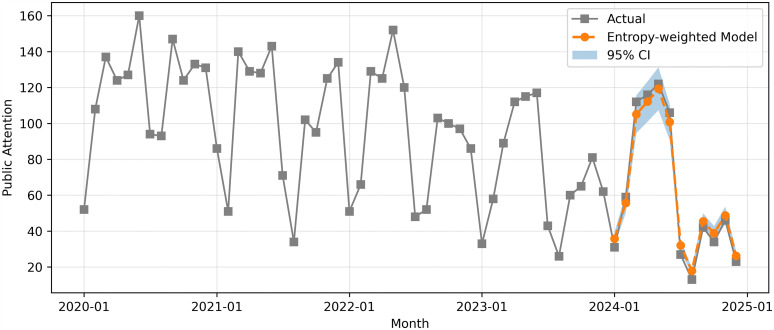
Entropy-weighted model forecasting with 95% CI.

The performance of entropy-weighted model forecast was further assessed by 95% confidence interval (CI) coverage rate, as shown in Fig 7.

The entropy-weighted model forecast closely followed the actual trend of PAEE and fit well with time series. In 2024, all observed values were in the range of model prediction, which was the 95% confidence range, showing that the model was stable and its future change range was reasonable. It should be noted that the CI coverage reflects the dispersion of ensemble forecasts rather than point-wise prediction accuracy. The CI analysis complements the point-forecast evaluation by illustrating the potential range of future variation, rather than implying superior predictive dominance.

## 5. Discussion

### 5.1. Interpretation of key findings

This study examines the evolution of PAEE in China from temporal, spatial, and modeling dimensions. The findings are consistent with previous studies, and improves our understanding of the development of public attention in the field of education.

PAEE in China shows a clear downward trend from 2016 to 2024. The monthly data also indicate a weak cycle and downward trend. Similar patterns have been found in studies on public attention to education, which often falls after some major polices [34,35]. With the adjustment of policies after the pandemic and the gradual easing of “mass entrepreneurship and innovation,” this decline in public attention appears reasonable. Previous studies also found downward trends regarding overall attention [38].

PAEE shows a clear “east high, west low” spatial pattern. The region’s concentration presents an enhancing trend. The Shannon entropy value decreased from 0.8912 to 0.7209. It means that the relatively balanced layout has gradually transformed into a more clustered structure. There is a trend of geographic polarization on the public discussion about education [39]. The Tsallis entropy shows the influence of leading provinces. Beijing and Guangdong share more information, showing a “center–periphery” pattern. It is similar to previous studies about attention to vocational education [33].

In forecasting results, the hybrid models have advantages over traditional single models in fitting accuracy, trend stability, and forecasting stability, reflecting the benefits of integrating structural and nonlinear components. This finding is consistent with previous studies emphasizing the complementary strengths of hybrid forecasting approaches [60,67]. The entropy-weighted model demonstrates competitive performance, particularly in terms of relative error stability and trend consistency. While its accuracy does not consistently exceed that of hybrid models, this approach effectively reduces extreme deviations, thereby enhancing robustness. The results confirm the validity of using entropy-based weighting integrated model [70]. It also confirms the idea that entropy weighting can reduce bias and enhance model robustness [69]. From a decision-oriented perspective, improvements in forecasting stability and directional consistency reduce the risk of premature or delayed policy responses, thereby enhancing the operational usefulness of the forecasts [14].

In general, after the early policy incentives ended, PAEE still presented a downward trend. Its spatial pattern shows a concentration and marginalization trend. It means there are clear differences in how social attention is distributed in different regions and different times. It shows uneven communication and a weak response to education policies, and a general downward trend in PAEE. The findings offer useful insights for public policy and educational governance. The decline in PAEE suggests that governments and education authorities should improve their communication and engagement. Regionally promotion activities and policy coordination should be strengthened. More digital media should be used to enhance public awareness and engagement. In addition, tracking changes in PAEE can also be used as a reference for policy adjustment. It helps maintain entrepreneurship education and narrow the differences in influence among regions.

### 5.2. Comparison with prior research

Based on previous research on entrepreneurship education, research topics have expanded to curriculum design, teaching innovation, learning evaluation, and policy formulation [21–23,72]. However, most of the research is still within the education system. They mainly focus on supply-side reforms, with little attention on public perception.

In contrast, this study takes a demand-side perspective and puts entrepreneurship education in a wider context of public understanding. We rebuild attention patterns by online search behavior and extend the existing research with a perspective based on public perception.

This study is beyond simple description or regression analyses. We apply entropy theory to explain complexity and concentration of spatial patterns. This method provides a clearer understanding of how structural changes emerge in different regional educational contexts. It also provides more temporal depth and structural information compared to studies that mainly applied single indicators such as education resources or economic level [73].

Some forecasting studies mainly applied single time series models such as ARIMA to forecast data for fixed periods [55]. Others used deep learning models like LSTM that function as black boxes [57]. These methods cannot make full use of different model advantages. To solve these problems, we developed a forecasting framework that combines multiple models and integrated them using entropy-based weighting. This method provides more flexibility to adjust trend estimation and error correction. It helps address the limitations of single models in generalization and stability.

Overall, this study is different from existing research in analytical perspective, methodology, and modeling strategy. These features form a distinctive framework that offers a reference for future research studies. We applied entropy theory and multi-model forecasting to build a data-driven and interdisciplinary framework that combines education, information science, and spatial analysis. This framework improved our understanding of public response and offered a new perspective to explore how social attention relates to policy outcomes and educational change.

### 5.3. Research significance

In terms of theoretical contribution, we introduced public attention into entrepreneurship education research from the demand side. Public attention is treated as an important indicator for assessing social responses. Compared to previous research mainly applied in fields that focus on curriculum design and teaching supply, our method promoted an interdisciplinary approach that involves education, communication, data analysis, and behavioral research.

Following recent work on actionable forecasting, this study evaluates forecasting performance in terms of its capacity to support data-informed governance decisions under uncertainty. The proposed forecasting framework helps education administrators track public interest dynamics, identify regions with low public attention, and improve the timing and targeting of policy delivery. By emphasizing stability and responsiveness, the framework supports more efficient resource allocation, enhanced public engagement, and greater educational equity.

Both Shannon entropy and Tsallis entropy are applied to describe spatial distribution and concentration of PAEE. It also develops an entropy-weighted model to impro.ve forecasting performance. The proposed framework provides a novel approach to study changing public attention patterns and offers a transferable method for data-driven research in the social sciences. Although this study is based on the Chinese context, its framework can serve as a reference for other countries to study public attention and education policy and practice.

### 5.4. Limitations and future research

Although this study provides a broad set of findings, it also has some limitations.

First, the analysis of PAEE is based on only one data source. Baidu is timely and widely used, but the exclusion of social media such as Weibo and Douyin may limit the range of PAEE patterns and cause behavioral differences. This limitation may also cause information bias. Since the data represent online behavior in China, the findings are context-specific, although the framework itself may be transferable to other settings.

Second, the study lacks integration of external contextual variables. While it focuses on the temporal evolution and spatial structure of PAEE, it has yet to systematically incorporate key background factors such as policy release timing, media coverage intensity, and major public opinion events. The omission of these aspects limits the analysis of the underlying drivers of shifts in attention and decreases the explanatory strength of the model overall, and particularly for sudden shifts.

Third, there is scope to enhance the model’s feature design. Although the fusion forecasting model demonstrates strong performance in terms of fit and stability, its input structure remains largely based on lagged historical variables, which may constrain generalizability when applied to different public attention domains or policy environments. From a methodological perspective, although the adopted validation protocol ensures a leakage-free out-of-sample evaluation, the forecasting performance is assessed based on a single temporal holdout. Future research may further examine the stability and robustness of ensemble performance using rolling-origin or multi-window validation schemes.

Considering these limitations, future work may follow several related themes. First, including multi-source data to develop a combined public attention index to reflect multiple sources of evidence of public engagement (search behavior and social relationship) [74]. Second, combining text mining and survey methods with context variables like policy releases, media coverage, and pathways for assigning attributes to aid the model in estimating the mechanisms underlying public attention [75]. Third, using new approaches such as causal inference to improve graph neural networks to advance live forecasting models where data structures are non-linear and dynamically shifting [76,77]. These extensions would contribute to building a more comprehensive and explanatory framework for public attention analysis.

## 6. Conclusion

Based on Baidu Index data and entropy theory, this study systematically examines the spatiotemporal dynamics of PAEE in China between 2016 and 2024, along with its forecasting models. The findings show that PAEE has a clear downward trend yearly, with weaker monthly changes. Spatially, PAEE changed from being more dispersed to more concentrated, with higher levels in eastern regions and lower levels in western provinces. In terms of forecasting, hybrid models generally outperform single models in error control and structural fitting, while the entropy-weighted model provides competitive performance by enhancing forecasting stability and robustness.

This study extends the theoretical understanding of entrepreneurship education by adding a public attention view. Meanwhile, it develops an entropy-based framework for analyzing and forecasting social behavior, which can be applied to similar data in other regions. The study provides a reference for practical work regarding public responses and offers some useful references for data-driven policy communication and resource allocation.

Since the data only come from the Baidu Index, the results are mostly constrained in China’s digital environment, and may contain some information bias. Future research may extend the data source to more platforms and add more context items, and use causal analysis to enhance the explanatory capacity and practical use of forecasting frameworks.
